# Mimicking urticaria: a Schnitzler syndrome case^☆^

**DOI:** 10.1016/j.abd.2023.10.004

**Published:** 2024-08-26

**Authors:** Kelielson Cardoso de Macêdo Cruz, Daniela de Abreu e Silva Martinez, Danielle Carvalho Quintella, Tullia Cuzzi, Sergio Duarte Dortas Junior, Solange Oliveira Rodrigues Valle

**Affiliations:** a Department of Immunology, Hospital Universitário Clementino Fraga Filho, Universidade Federal do Rio de Janeiro, Rio de Janeiro, RJ, Brazil; b Department of Pathology, Hospital Universitário Clementino Fraga Filho, Universidade Federal do Rio de Janeiro, Rio de Janeiro, RJ, Brazil

Dear Editor,

Schnitzler Syndrome (SchS) is a rare disorder, with ∼350 cases reported in the literature, characterized by a neutrophilic urticarial dermatosis and monoclonal gammopathy (IgM in more than 90% of the cases), associated with clinical and biological signs of inflammation.^1^, ^2^, ^3^ In 2013, Lipsker, Schnitzler, and other experts met and proposed the Strasbourg diagnostic criteria, which are widely used today to diagnose SchS (Table 1).^1^, ^4^ We report a case of a man with a clinical and laboratory diagnosis of SchS in Brazil.

**Table 1:** Strasbourg diagnostic criteria of Schnitzler syndrome.

Obligate criteria
- Chronic urticarial rash and
- Monoclonal IgM or IgG
Minor Criteria
- Recurrent fever^a^
- Objective findings of abnormal bone remodeling with or without bone pain^b^
- A neutrophilic dermal infiltrate on skin biopsy^c^
- Leukocytosis and/or elevated CRP^d^
Definite diagnosis if
- Two obligate criteria AND at least two minor criteria if IgM and three minor criteria if IgG
Probable diagnosis if
- Two obligate criteria AND at least one minor criteria if IgM and two minor criteria if IgG

a Must be >38 °C and otherwise unexplained. Occurs usually ‒ but not obligatory ‒ together with the skin rash.

b As assessed by bone scintigraphy, MRI, or elevation of bone alkaline phosphatase.

c Corresponds usually to the entity described as “neutrophilic urticarial dermatosis”; absence of fibrinoid necrosis and significant dermal edema.

d Neutrophils >10,000 mm^3^ and/or CRP > 3 mg/dL.

A 67-year-old man presented to our department with recurrent pruritic, not painful, urticaria-like lesions on the extremities and the trunk (Fig. 1). The lesions resolved within 24 hours without leaving behind dusky hyperpigmentation. He had intermittent fever of up to 39 °C, generalized arthralgia, and fatigue. The inflammatory episodes lasted for 2–5 days with severe general impairment. The patient suffered outbreaks with variable intensity on almost a monthly basis over the past fifteen years. Histopathology of lesional skin showed a perivascular and interstitial polymorphonuclear infiltrate, described as consistent with the diagnosis of urticarial lesions and compatible with SchS (Fig. 2).

**Fig. 1:**
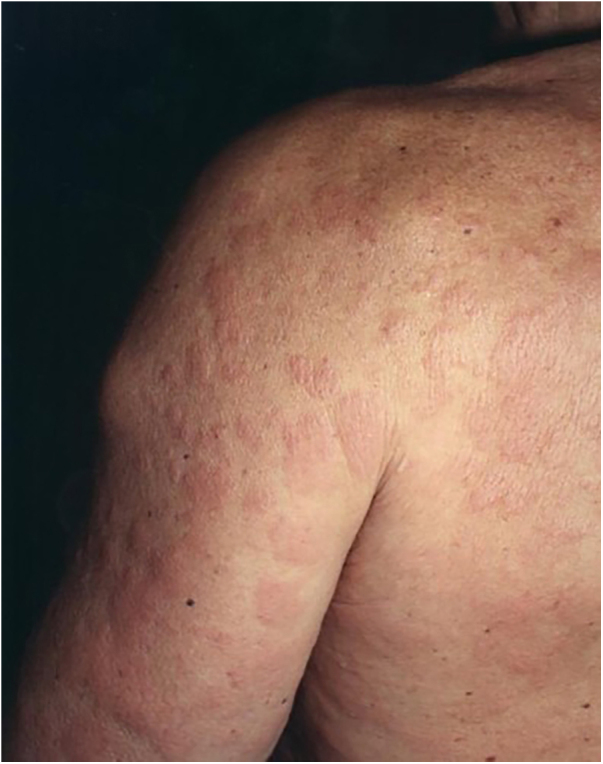
Urticarial lesions on the trunk and left arm.

**Fig. 2:**
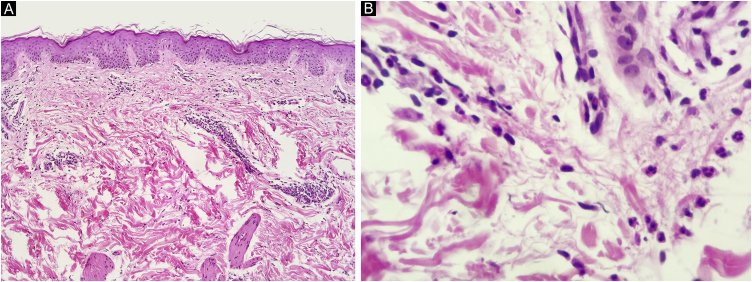
Inflammatory cells around superficial dermal vessels with neutrophils (A, Hematoxylin & eosin, ×100) also observed in the interstitial space among collagen fibers (B, Hematoxylin & eosin, ×400).

Laboratory investigations revealed leucocytosis (up to 21.000 mm^3^, ref 4000–11600 mm^3^), an elevated ESR (120 mm/hr; 0∼20 mm/hr), and increased IgM levels (2550 mg/dL; 46∼260 mg/dL) in serum protein electrophoresis (Fig. 3). In the bone scan, there is a slight asymmetry of uptake in the tibias, slightly greater on the left. Bone marrow biopsy was performed with a negative cytogenetic study for lymphoproliferative diseases. Based on these clinical and laboratory findings, he was diagnosed with Schnitzler Syndrome.

**Fig. 3:**
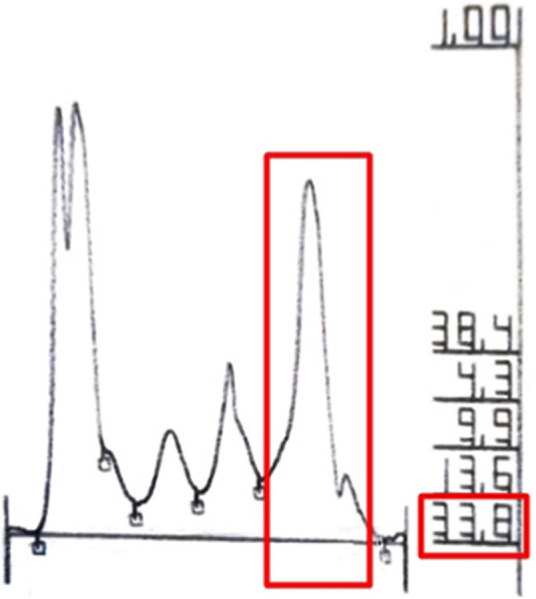
Increased IgM levels in serum protein electrophoresis test.

He was started on a high-dose corticosteroid, NSAIDs, and antihistamines with partial remission of his symptoms.

There are a few cases reported in Latin America and in general, it is underdiagnosed despite the well-established diagnostic criteria, but the pathogenesis is unknown.^5^

The major complication is hematological malignancy, with lymphoproliferative disorder occurring in about 10%‒20% of patients.^5^, ^6^ In addition to chronic spontaneous urticaria, other differential diagnoses should be considered (Table 2).^7^

**Table 2:** Differential diagnoses of Schnitzler syndrome.

Autoimmune diseases
Adult Onset Still Disease (AOSD)
Systemic Lupus erythematosus
Acquired C1-esterase inhibitor deficiency
Hematologic diseases
Monoclonal gammopathy of undetermined significance (MGUS)
POEMS syndrome (polyneuropathy, organomegaly, endocrinopathy, mono-clonal gammopathy and skin changes)
Waldenström macroglobulinemia
Lymphomas
Multiple myeloma
Hereditary autoinflammatory syndromes
Cryopyrin-associated syndromes (CAPS):
Familial cold urticaria
Muckle-Wells syndrome
Chronic infantile neurologic cutaneous and articular syndrome (CINCA)
Infectious diseases
Hepatitis B and C
Chronic meningococcemia
Other
Chronic Spontaneous Urticaria
Hypocomplementemic urticarial vasculitis
Delayed pressure urticaria
Cryoglobulinemia
Behçet syndrome
Mastocytosis

At present, for patients with CRP < 3 mg/dL, treatment options include, colchicine, Nonsteroidal Anti-inflammatory Drugs (NSAIDs), and hydroxychloroquine.^1^, ^2^ Corticosteroids has a moderate effect and antihistamine therapy has no effect.^8^ According to Simon et al., the efficacy of colchicine is only 25%, but based on the benefit/risk ratio, colchicine is recommended as the first choice of treatment.^9^ Experts recommend the use of anakinra (IL-1 block) in more symptomatic patients, such as Erythrocyte Sedimentation Rate (ESR) and CRP above the upper limit of normal (CRP > 3 mg/dL).^1^

Treatment with corticosteroids, NSAIDs, and antihistamines is symptomatic and unsatisfactory.^9^ Anakinra (IL-1-neutralizing) is the choice drug. The effect of inhibition of IL-1 has led to new expectations, but there is currently unavailable in Brazil. An alternative drug could be canakinumab, a human anti-IL-1β monoclonal antibody aiming at the neutralization of 1β signaling.^10^

In patients presenting with chronic urticaria associated with signs of systemic inflammation, this rare and debilitating syndrome should be considered. Early treatment can improve patient's quality of life and disease prognosis.

## Financial support

None declared.

## Authors’ contributions

Kelielson Cardoso de Macêdo Cruz: Preparation and writing of the manuscript; intellectual participation in propaedeutic and/or therapeutic management of studied cases; approval of the final version of the manuscript; critical literature review.

Daniela de Abreu e Silva Martinez: Preparation and writing of the manuscript; approval of the final version of the manuscript.

Danielle Carvalho Quintella: Preparation and writing of the manuscript; approval of the final version of the manuscript.

Tullia Cuzzi: Preparation and writing of the manuscript; approval of the final version of the manuscript.

Sergio Duarte Dortas Junior: Preparation and writing of the manuscript; intellectual participation in propaedeutic and/or therapeutic management of studied cases; approval of the final version of the manuscript.

Solange Oliveira Rodrigues Valle: Preparation and writing of the manuscript; intellectual participation in propaedeutic and/or therapeutic management of studied cases; approval of the final version of the manuscript.

## Conflicts of interest

None declared.
